# Genome sequence of a native nitrogen-fixing *Bradyrhizobium sp*. strain RCAM1614 isolated from lupine nodules

**DOI:** 10.1128/mra.01048-25

**Published:** 2025-11-04

**Authors:** Eugenia Yu. Denisova, Evgenii A. Kirichek, Viktor E. Tsyganov

**Affiliations:** 1 Saint Petersburg State University, Saint Petersburg, Russia; 2 Laboratory of Molecular and Cell Biology, All-Russia Research Institute for Agricultural Microbiology (ARRIAM) 117473 , Saint Petersburg, Russia; Rochester Institute of Technology, Rochester, New York, USA

**Keywords:** legume-rhizobial symbiosis, symbiotic nodules, *Bradyrhizobium barranii*

## Abstract

This report describes the genome sequence of *Bradyrhizobium* sp. strain RCAM1614, which was isolated from the root nodules of *Lupinus angustifolius* L. in the Leningrad district of Russia. The genome has a size of 9.77 Mbp and shows 96.1% completeness, which enables precise taxonomic classification.

## ANNOUNCEMENT

Species of the genus *Bradyrhizobium* are recognized as important nitrogen-fixing symbionts of various legumes, including lupine, groundnut, and soybean (1–3). Strain RCAM1614 was isolated according to Vincent (4) approximately 20 years ago from effective nodules of *Lupinus angustifolius* L. in the Leningrad district, Russia and deposited into the network collection of bioresources in the field of genetic technologies for agriculture (RCAM) as *Bradyrhizobium* sp.

Strain was preserved in yeast extract mannitol (YEM) (5) medium with 40% glycerol at −80°C. For DNA isolation, strain was cultivated on YEM at 28°C for 5 days. Genomic DNA was extracted using the Monarch Genomic DNA Purification Kit T3010L (New England Biolabs, USA) according to the manufacturer’s recommendations without size selection.

Oxford Nanopore Technologies (ONT) sequencing was carried out in the All-Russia Research Institute for Agricultural Microbiology (ARRIAM). Sequencing libraries were prepared using the Native Barcoding Kit 24 V14 (SQK-NBD114.24) (Oxford Nanopore, United Kingdom) according to the manufacturer’s instructions, skipping the DNA-shearing step, and sequenced on a MinION (Oxford Nanopore) device with FLO-MIN114 flow cell. Basecalling was performed with Dorado v1.0.1 (https://github.com/nanoporetech/dorado/) using the dna_r10.4.1_e8.2_400bps_sup@v5.2.0 model. Raw read quality control was performed with NanoPlot (6) v1.44.1. In total, 285,977 reads with N_50_ = 6,796 bp were obtained.

The genome was assembled *de novo* using Flye (7) v2.9.6 with the (--nano-raw) option, followed by polishing with Medaka v1.7.2 (https://github.com/nanoporetech/medaka). Assembly quality was assessed with QUAST (8) v5.3.0 and completeness was evaluated with BUSCO (9, 10) v5.8.0 using the rhizobiales_odb10 data set. The genome assembly was deposited at GenBank and annotated using the NCBI Prokaryotic Genome Annotation Pipeline (11) v6.10. Default parameters were used for all listed software unless otherwise noted.

The draft genome consists of 5 contigs, with a total length of 9,773,268 bp, a GC content of 63.48%, an assembly N_50_ value of 9,330,649 bp, an estimated coverage of 112×, and a completeness of 96.1%. It contains 9,089 protein-coding genes, 55 tRNAs, and 6 rRNAs.

**Fig 1:**
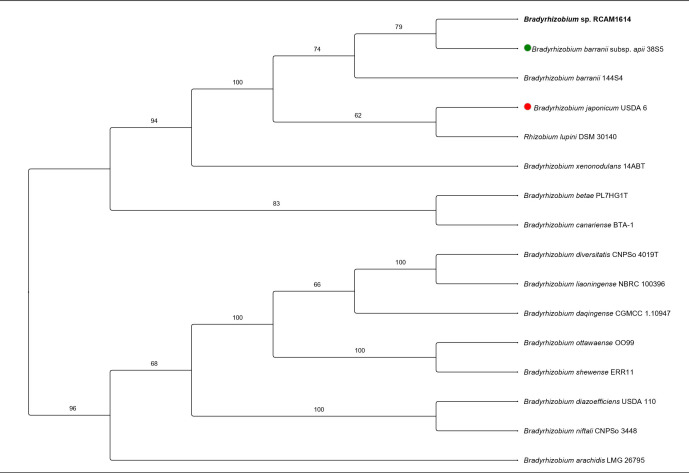
*Bradyrhizobium* sp. RCAM1614 whole-genome based phylogenetic analysis. The numbers above the branches are the GBDP pseudo-bootstrap support values greater than 60%. Strain RCAM1614 (bold), *B. barranii* subsp. *apii* 38S5ᵀ (green dot), *B. japonicum* USDA 6ᵀ (red dot).

Primary taxonomic identification was performed using the Type (Strain) Genome Server (TYGS) (12, 13) v401 platform. Phylogenomic analysis based on whole-genome sequences using the Genome BLAST Distance Phylogeny (GBDP) method placed strain RCAM1614 within a well-supported clade alongside *Bradyrhizobium barranii* strains. The most closely related type strain was *B. barranii* subsp. *apii* 38S5ᵀ with a digital DNA-DNA hybridization value of 75.1% (Fig. 1). The final taxonomy check was provided by GenBank with *B. barranii* 97.36% average nucleotide identity match.

## Data Availability

Assembly and sequence data have been deposited in the GenBank under the BioProject accession number PRJNA1311437. Assembly has been deposited under the accession number GCA_052402455.1. The demultiplexed FASTQ file, with barcodes removed, from the MinION run has been deposited in the Sequence Read Archive (SRA) under number SRX30289055. This announcement describes the first version of the genome assembly.
